# Load-Balancing of Kubernetes-Based Edge Computing Infrastructure Using Resource Adaptive Proxy

**DOI:** 10.3390/s22082869

**Published:** 2022-04-08

**Authors:** Quang-Minh Nguyen, Linh-An Phan, Taehong Kim

**Affiliations:** School of Information and Communication Engineering, Chungbuk National University, Cheongju 28644, Korea; quangminh@cbnu.ac.kr (Q.-M.N.); linhan@cbnu.ac.kr (L.-A.P.)

**Keywords:** containerization, edge computing, Kubernetes, kube-proxy, load-balancing, metric-server, microservice

## Abstract

Kubernetes (K8s) is expected to be a key container orchestration tool for edge computing infrastructures owing to its various features for supporting container deployment and dynamic resource management. For example, its horizontal pod autoscaling feature provides service availability and scalability by increasing the number of replicas. kube-proxy provides traffic load-balancing between replicas by distributing client requests equally to all pods (replicas) of an application in a K8s cluster. However, this approach can result in long delays when requests are forwarded to remote workers, especially in edge computing environments where worker nodes are geographically dispersed. Moreover, if the receiving worker is overloaded, the request-processing delay can increase significantly. To overcome these limitations, this paper proposes an enhanced load balancer called resource adaptive proxy (RAP). RAP periodically monitors the resource status of each pod and the network status among worker nodes to aid in load-balancing decisions. Furthermore, it preferentially handles requests locally to the maximum extent possible. If the local worker node is overloaded, RAP forwards its requests to the best node in the cluster while considering resource availability. Our experimental results demonstrated that RAP could significantly improve throughput and reduce request latency compared with the default load-balancing mechanism of K8s.

## 1. Introduction

Containerization technology is a promising solution for building edge computing infrastructures for smart cities, buildings, and grids [1,2] because of its numerous benefits such as inherent fault isolation, high security, portability, and lightweightness [3,4,5]. However, to efficiently deploy containers and manage application resources in an edge computing environment [6,7], a container orchestration tool that has resource allocation and utilization, scalability, high availability, and load-balancing functions, is also essential [8,9].

Among the various container orchestration tools on the market, Kubernetes (K8s) is the most popular [10]. K8s provides many of the important features listed above and is suitable for container-based edge computing infrastructure [11]. Its various features facilitate the rapid deployment of applications (services) without complicated configuration and installation steps. For example, each container of an application can be deployed as multiple replicas (pods) across worker nodes in a K8s cluster to provide high availability for the application. Meanwhile, the autoscaling feature in K8s can dynamically upscale and downscale the number of replicas according to the current workload of the application [12,13]. Although the number of pods is often changed in a K8s cluster, all requests to a particular application are forwarded to its appropriate backend pods by a K8s component called *kube-proxy* that is installed in every node. Consequently, the operation of *kube-proxy* can directly affect the performance of the application (e.g., throughput and latency) in a K8s cluster.

The *kube-proxy* essentially operates as a network load balancer by evenly distributing client requests to each pod of an application. However, this strategy has several disadvantages, especially in edge computing environments. First, because the pods of an application can be geographically dispersed and there can be delay among worker nodes [14], forwarding requests across nodes can cause significant delays. Second, forwarding requests without considering the resource status of the receiving nodes can produce non-optimal results. For example, if a receiving node is overloaded or has a high network latency, the requests will be significantly delayed, resulting in dramatically decreased application performance.

To overcome the limitations outlined above, this paper proposes an enhanced *kube-proxy* called *resource adaptive proxy* (*RAP*). The main idea behind *RAP* is to forward as many client requests as possible to pods located at a local node for minimizing request-handling delays. If a local node becomes overloaded, *RAP* forwards requests to the best remote node in the cluster. The best remote node is selected based on the availability of computational resources and the network latency between local and forwarded nodes. If all of the remote nodes are overloaded, *RAP* does not forward the requests but handles them locally to avoid further congestion from the forwarding. The main contributions of this study are as follows:

- We address the problem of the default load-balancing operation in K8s that cause long network delays and degrade the overall throughput in edge computing environments where the edge nodes are geographically distributed;
- To solve the aforesaid problem, we propose a novel approach called *RAP* that has two main features. First, *RAP* monitors the resources of each pod and the round-trip delay among worker nodes in the cluster required to perform resource adaptive load-balancing. Second, *RAP* handles requests locally as much as possible to improve the latency and throughput, and it redirects requests to remote worker nodes only when local nodes are overloaded;
- We conducted extensive evaluations that demonstrated the limitations of the default *kube-proxy* and the benefits of the proposed *RAP* algorithm in a K8s-based edge computing environment. The experimental results proved that, compared to default *kube-proxy* modes, RAP significantly improves the overall throughput and maintains the latency at a sufficiently low level to be suitable for latency-sensitive applications.

The remainder of this paper is organized as follows. Section 2 presents related work. Section 3 briefly explains the architecture and operation of K8s. Section 4 describes in detail the architecture, algorithm, and workflow of *RAP*. Section 5 outlines the conducted performance evaluations and provides an analysis of the obtained results. Section 6 presents concluding remarks.

## 2. Related Work

The edge computing paradigm was proposed to reduce latency between end devices and cloud servers, ultimately increasing both the quality of service (QoS) and quality of experience (QoE) [15] for users. Many methods have been proposed to exploit this benefit of edge computing. For example, Abouaomar et al. [16] proposed an algorithm that chooses the most optimal edge node to handle as much latency-sensitive application traffic as possible. The optimal edge node is selected based on four resource parameters: CPU, storage, memory, and networking. Phan et al. [17] proposed a dynamic offloading solution for when an edge node becomes overloaded to reduce latency and increase the performance of software defined networking (SDN)-based edge computing environments. Because SDN can fully control the network rules among edge nodes, it can choose the best nodes to offload the requests to overloaded nodes.

Regarding the development of applications for edge computing environments, recent studies [6,9] have analyzed the advantages of using containerization technology and microservice architecture. Khan [9] argued that because each microservice-based application could be composed of hundreds of containers, a container orchestration tool such as K8s is essential to manage the containers in a cluster. Nguyen et al. [18] provided a thorough explanation of the concepts underlying K8s, including the key components such as horizontal pod autoscaling that provides application scalability, the *scheduler* that arranges the ratio of application pods on worker nodes, and a special component called metric-server. Furthermore, they elucidated the application of the K8s platform in the edge computing architecture. However, they did not address the feasibility and limitations of K8s-based edge computing infrastructures. Kayal [19] conducted experiments to evaluate the feasibility and industrial practicality of K8s-based edge computing infrastructure. Their results indicated that K8s has critical performance limitations for clusters. Consequently, they modified the *scheduler* component of K8s to improve performance and reduce communication costs.

Abouaomar et al. [16] evaluated the performance of K8s-based edge computing for latency-sensitive applications. They found that the performance was deficient; consequently, they proposed an improvement to the K8s *scheduler* that prioritized deploying pods on edge nodes with high bandwidth. Their proposed *scheduler* significantly reduced the network latency and time for scheduling and deploying new pods. Santos et al. [20] proposed the Service Function Chain (SFC) controller that is an extension of the scheduling features of the default K8s. The SFC controller makes an allocation decision based on bandwidth resources between worker nodes to optimize resource allocation in a cluster and reduce the end-to-end latency. They reported that the SFC controller reduced the latency by up to 18% compared to the default *scheduler* algorithm. However, the configuration steps of SFC controllers are complicated, leading to increased scheduling time. Toka [21] proposed an advanced edge *scheduler* based on the default *scheduler* mechanism of K8s. Their solution provided high reliability for edge computing services and ensured that latency requirements were met. Further, their experimental results showed that the scheduling time was reduced by over 40% compared to the default K8s *scheduler*. However, the new *scheduler* algorithm used over 10% of system resources to make allocation decisions, and the algorithm complexity was $O(|N{|}^{2}+|P{|}^{2})$.

Wojciechowski et al. [22] proposed a novel approach to K8s pod scheduling called NetMARKS. They used Istio Service Mesh to improve K8s scheduling by exploiting dynamic network metrics. Similarly, Nguyen et al. [23] found that the default load-balancing mechanism of the *kube-proxy* component in K8s caused a high delay in processing requests in a K8s-based edge computing environment. This is because *kube-proxy* evenly distributes the requests to all pods of an application, regardless of the current location and resource status of the pods. To address this limitation, they proposed ElasticFog [23] to dynamically allocate application pods based on the network traffic distribution. In particular, ElasticFog deploys more pods on edge nodes that receive more requests. Note that ElasticFog does not interfere with the load-balancing mechanism of K8s; it only changes the locations of pods in the cluster. However, as edge node resources are limited, deploying many pods on a local node may not always be feasible.

Phuc et al. [24] proposed the traffic-aware horizontal pod autoscaler (THPA) that upscales and downscales application pods according to the amount of network traffic accessing the cluster. Their evaluation results indicated that THPA improves the throughput of the system and reduces the latency compared to the default horizontal pod autoscaler (HPA) feature of K8s. However, THPA has a response time issue during the autoscaling process, i.e., because their proposed mechanism depended on HPA, if the traffic suddenly increased at a node, delays would occur before autoscaling could be applied.

Existing studies have predominantly provided solutions that reduce latency by focusing on changing the algorithms of the *scheduler* component. However, latency significantly occurs during load-balancing when requests are being forwarded between workers in a K8s cluster. Therefore, we propose a load-balancing mechanism that ensures that requests are handled locally to the maximum extent possible and forwards requests to the best node by exploiting the resource status of the nodes. Our proposed approach can effectively load-balance and adapt to requests from clients in container-based edge computing.

## 3. Preliminaries of Kubernetes

### 3.1. Kubernetes Architecture

**Figure 1:**
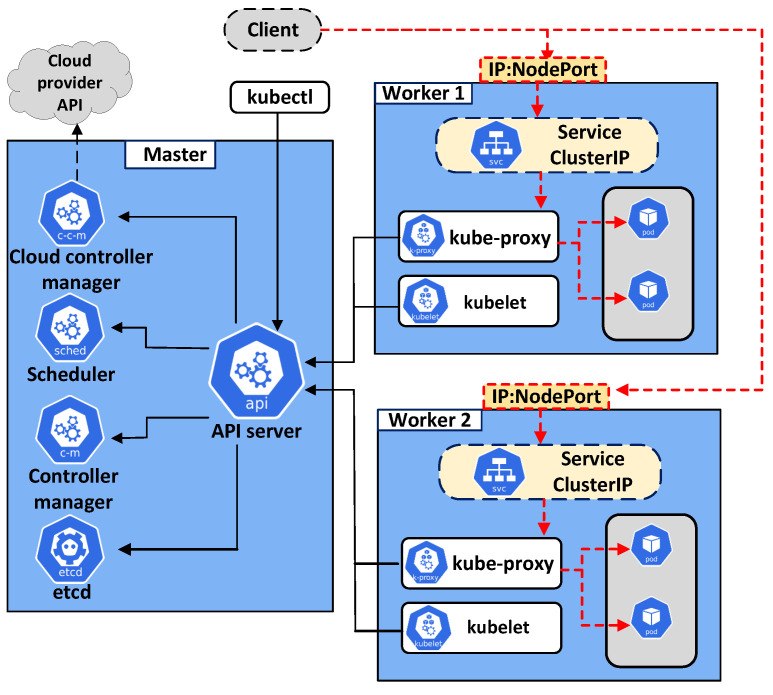
Kubernetes architecture.

K8s is an open source project for container orchestration that facilitates deployment, management, and autoscaling in the operation and services of container-based applications [18]. As shown in Figure 1, a K8s cluster consists of a master node and worker nodes. In K8s, applications run in pods: a pod is the smallest unit containing one or more containers. The master node, as the controller of a K8s cluster, detects cluster issues and makes cluster management decisions [23]. It consists of four essential components: *etcd, kube-scheduler, kube-controller manager,* and *kube-apiserver* [25]. The function of each master node component is described below [26]:

- *etcd*: This is the key data storage component of a K8s cluster. It saves all events that occur in a cluster except for the application data;
- *kube-scheduler*: This is responsible for finding the best node to deploy a new pod. A suitable node is selected based on several criteria, such as the resource request by the pod, available node resources, and *affinity* and *anti-affinity* rules [27];
- *kube-apiserver*: Through *kube-apiserver*, an administrator can communicate with a K8s cluster using the *kubectl* command-line tool. Moreover, *kube-apiserver* has a connection to worker nodes to manage pod operations through the *kubelet* and *kube-proxy* tools;
- *kube-controller-manager*: This is a critical monitoring loop of the cluster. *kube-controller-manager* monitors the current state of the cluster—which must match the desired state—and adjusts resources to make the current state close to the designed state.

In a K8s cluster, the application pods are deployed and executed on the worker nodes [28]. A worker node has three main components: *kubelet, kube-proxy,* and *container runtime* [29]. The roles of these components are as follows:

- *kubelet*: This component has a connection to *kube-apiserver* of the control plane to receive commands and return status reports about pods and worker nodes;
- *kube-proxy*: This is an essential component of each worker node. It maintains the connection between application pods and load-balancing requests in the cluster;
- *container runtime*: This component is used to execute containers and manage the container images on each worker node. The container runtime must be installed on each node to deploy pods.

K8s allows users to access pods in a cluster through an abstract object called *Service* instead of allowing direct access to the pods because the IP address of a pod is changed if the pod is restarted or newly deployed [30]. *Service* exposes a set of related pods and allows both internal and external connections. When *Service* is initialized, it is assigned a virtual IP called *ClusterIP* that is not to be changed unless the *Service* object is removed. Clients can access the services of an application through *workerIP:NodePort*. The *Service* object acts as a middle connection layer between outside clusters and application pods. It creates internal connections from *NodePort* to *Service:port* and another connection from *Service:port* to suitable application containers running inside pods with the PodIP:containerPort pair, known as endpoints. After finishing the connection initialization process, the *kube-proxy* component is assigned to load-balance requests in the cluster.

### 3.2. kube-proxy

**Figure 2:**
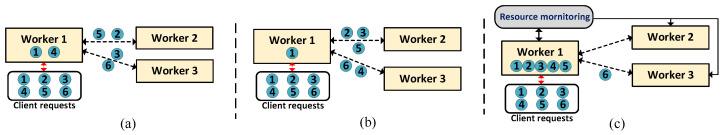
Load-balancing algorithms of the default *kube-proxy* and *RAP*: (**a**) *round-robin*, (**b**) *random*, and (**c**) *RAP*.

To provide highly scalable and available applications, it is possible to deploy multiple replicas (pods) for a container. Therefore, *kube-proxy* can be used to load-balance requests for appropriating backend pods of the application. There are three operation modes in *kube-proxy*: *userspace*, *iptables*, and *IPVS* [30]. The *userspace* mode uses a round-robin algorithm [30] to select suitable endpoints, as shown in Figure 2a. It offers many flexible customizations in the algorithm because it allows developers to add more features by modifying the source code. However, *userspace* has a bottleneck limitation in that it must switch and copy data between the kernel and user spaces. Meanwhile, *iptables* mode works in the kernel space of a Linux system, so it installs connection rules in iptable space without the control of the K8s control plane. The *iptables* mode randomly chooses endpoints via Linux iptable rules [30], as shown in Figure 2b. Finally, *IPVS* is the newest proxy mode of K8s; it works in the kernel in a manner similar to *iptables*. However, the connection between *Service* and endpoints is created according to a special *IPVS* algorithm and is built based on the netlink interface. The *IPVS* mode supports several load-balancing algorithms such as round-robin, slightest connection, destination hashing, source hashing, shortest expected delay, and never queue. In the default balancing algorithm, the backend pods are selected using a round-robin algorithm.

Although *kube-proxy* modes provide different balancing algorithms to improve the performance of applications in K8s-based edge computing architecture [31], it does not offer an examination feature for checking the hardware status of the neighboring workers and the quality of the connection between workers. In addition, we found that the throughput of the cluster is decreased if requests are forwarded to an overloaded worker and request delays increase when requests are forwarded to a worker node that has high latency. These limitations can have negative effects on sensitive applications in edge computing environments. Therefore, we propose *RAP* to maximize throughput and minimize latency. As shown in Figure 2c, our algorithm aims to handle all requests at local nodes. If a local node becomes overloaded, *RAP* finds the best nodes based on resource status and connection delay to forward requests.

## 4. Resource Adaptive Proxy (RAP)

### 4.1. Kubernetes-Based Edge Computing Architecture

**Figure 3:**
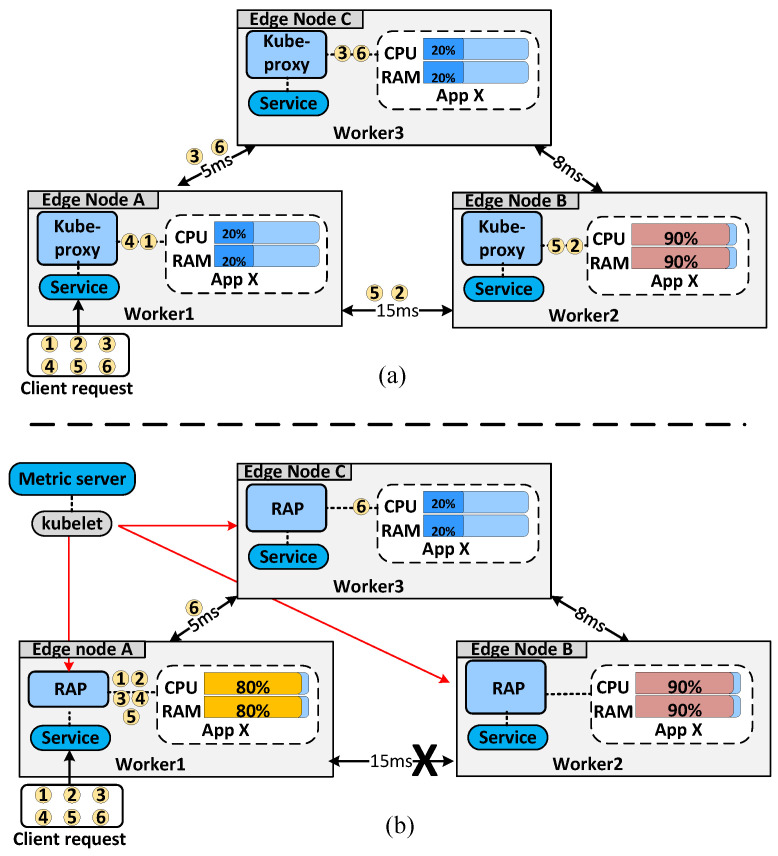
Load-balancing algorithms of (**a**) *userspace* and (**b**) *RAP* in K8s-based edge computing architecture.

As illustrated in Figure 3, K8s can be applied to manage microservice-based applications and improve the QoS of services deployed in edge computing infrastructures [5,32]. The key benefits include the multifariousness of services, ease of application deployment, load-balancing of requests, autoscaling, and fault tolerance [33]. Based on these advantages, end-user devices can access the services of applications that run on edge nodes. To continuously supply service to the Internet of Things (IoT) devices of end users, edge computing infrastructure is built to cover a large area, such as a smart city or intelligent transportation [34,35,36]; thus, edge nodes are located far from each other. It is also important to note that the behavior of users accessing a service is dynamic, so the number of requests in an edge computing environment varies over time and location [37].

K8s provides the *kube-proxy* load-balancing component. When users send requests to an edge node, *kube-proxy* distributes them evenly to backend pods, as shown in Figure 3a. For example, as shown in Figure 3a, we set up a cluster containing two available workers (edge nodes A and C) and an overloaded worker (edge node B). All worker nodes used the default load-balancing algorithm of K8s, and there was a network delay between worker nodes. When a client sent six requests to Worker 1, only two requests were processed locally; the remaining four requests were split equally between Workers 2 and 3. We can see that forwarding Requests ➁ and ➄ to Worker 2 was not an optimal decision because the network connection delay between Workers 1 and 2 was the highest in the cluster. Therefore, long delays were inevitable, owing to the round-trip time between edge nodes when we applied the default kube-proxy. In particular, if requests are forwarded to overloaded nodes such as Worker 2, the delay will increase significantly.

Our proposed *RAP* algorithm solves this problem, as shown in Figure 3b. In contrast to the default *kube-proxy*, *RAP* preferentially handles requests at a local worker node. If the local worker becomes overloaded after handling five requests, the remaining request (Request ➅) is forwarded to the best worker to ensure maximum throughput. The detailed design of *RAP* is presented in the following subsection.

### 4.2. RAP Workflow and Algorithm

In this section, we describe in detail the workflow and algorithm of *RAP* that runs on all worker nodes of a K8s cluster. *RAP* periodically collects the resource statuses of the CPU and RAM as well as the network delay between the edge nodes to make optimal load-balancing decisions for a K8s cluster. The goal of *RAP* is to minimize request latency and improve the overall throughput.

The operation of *RAP* in a K8s-based edge computing architecture is illustrated in Figure 3b. When users send requests to an edge node in a cluster, *RAP* considers the application resources in that edge node when making a load-balancing decision. The requests are preferentially handled by the local nodes. If the local node is overloaded, the requests are forwarded to the best edge node to minimize the delay of requests. As shown in Figure 3b, most requests are handled internally by edge node A and only some requests are forwarded to edge node C. This indicates that the application resources on edge node A are all in use; therefore, the *RAP* in edge node A forwards a portion of the requests to edge node C. Edge node C is chosen by edge node A because it has sufficient computational resources and the network delay between them is less than that of other nodes, as illustrated in Figure 3a. To provide the resource data of worker nodes to the *RAP* algorithm, we utilized a default resource monitoring tool of K8s called *metric-server* [38]. This is a lightweight component that can monitor the resource metrics of the endpoint such as CPU and RAM usage with only one *millicore* CPU and two *mebibytes* of RAM [38]. The delay value is collected and converted to combine with other resource parameters such as CPU and RAM to make optimal load-balancing decisions.

The procedure used in *RAP* is outlined in Algorithm 1. On each worker node, *RAP* runs a function to collect and update the endpoints of the node (i.e., backend pods) periodically in Lines 2–9. *RAP* initializes a mechanism that frequently updates the endpoints list of an application because it can be changed by the endpoint addition or removal event, or the autoscaling operation. Then, endpoints are categorized into different subendpoint lists according to each worker node. Through the *metric-server* component and latency measurement tool, *RAP* collects and uses endpoint resources and then selects the remote node among available nodes in the cluster in Lines 10–24. Because the measurement units of CPU, RAM, and latency differ, their parameters must be normalized using a common denominator for *RAP* to calculate the resource score of each worker node. The best node is selected as the node that has the highest resource score.

Normalizing and scoring are carried out as follows. As summarized in Table 1, *r* and *N* denote the resources of application α and the set of worker nodes containing the pods of α, respectively. $R_{r}^{i}$ denotes the specific resource *r* at worker node *i*, which can be a hardware resource (e.g., CPU or RAM) or network delay. $\beta_{r}^{i}$ is the individual denominator used in the normalization process, and $\phi_{r}^{i}$ is the result of the normalization process of resource *r* at worker node *i*. In addition, note that $W_{r}$ denotes the weight factor of resource *r*. $\tau_{i}$ and $\tau_{BestNode}$ are the scores of individual node *i* and the best node, respectively. It is important that the candidates of the remote node contain endpoints of the application α and sufficient resources to satisfy the requirement. Moreover, each resource type *r* has a special unit, such as *millicore* for CPU and *mebibytes* for RAM. Therefore, the units of individual resources specified by each candidate *i*, $R_{r}^{i}$, must be normalized to a common unit before calculating the resource score for the candidate, as defined by Equation (1):

(1) $$\phi_{r}^{i}=\frac{\left(R_{r}^{i}\right)}{\beta_{r}^{i}}.$$

**Algorithm 1** Resource Adaptive Proxy (RAP)/* Define global variables*/***BestNode***                           ▹ Most powerful resource remote worker***EndpointsMap***                           ▹ List of endpoints of each endpoint***NextEndpoints***                           ▹ Endpoints used in the load-balancing***ResLists***                        ▹ Resource list including CPU, RAM, and latency  1:**function**ResourceAdaptiveProxy  2:  /* Create separate endpoint list based on name of node*/  3:  CurrentEndpoints=GetAppInfo(app).endpoints  4:  **if** CurrentEndpoints≠LastestEndpoints **then**  5:    **for** *endpoints, node* **in** *GetAppInfo(app).Item[]* **do**  6:      EndpointsMap[node].add(endpoints)  7:    **end for**  8:    LastestEndpoints=CurrentEndpoints  9:  **end if**10:  /* Calculate resources and return the best remote node*/11:  MetricServer.Initialize()12:  **for** *node* **in** *GetAppInfo(app).Item[]* **do**13:    AppRes[node].cpu←MetricServer.ResLists.CPU14:    AppRes[node].ram←MetricServer.ResLists.RAM15:    //Calculate score by summing normalized resource.16:    **if** node≠GetHostname() **then**17:      **if** AppRes[node].cpu<Threshold**&&**AppRes[node].ram<Threshold **then**18:        $Score\left[node\right]=\sum_{r\in Resources}W_{r}*(Normalize\left(AppRes\left[node\right]\right)$19:        **if** Score[node]>HighestScore **then**20:          HighestScore=Score[node]21:          BestNode←node22:        **end if**23:      **end if**24:    **end if**25:  **end for**26:**end function**27:**function**Loadbalancer28:  /*Incoming requests are forwarded to NextEndpoint.*/29:  **if** IncomingRequest≠Null **then**30:    LocalNode←GetHostname()31:    **if** AppRes[LocalNode].cpu<Threshold **&&** AppRes[LocalNode].ram<Threshold **then**32:      NextEndpoint←EndpointsMap[LocalNode]33:    **else**34:      NextEndpoint←EndpointsMap[BestWorker]35:    **end if**36:    //Forward Incoming Request to NextEndpoint37:  **end if**38:**end function**

By adjusting the ratio of $W_{r}$ according to the specifications of the application, the score $\tau_{i}$ of each candidate *i* can be calculated by summing the normalized value $\phi_{r}^{i}$ according to Equation (2), and the best worker node can be chosen with the highest score according to Equation (3):

(2) $$\tau_{i}=\sum W_{r}\phi_{r}^{i},$$

(3) $$\tau_{BestNode}=max\left(\tau_{i}\right).$$

In summary, the *RAP* function returns two essential values: an endpoint map that includes a collection of key–value pairs as locations and IP addresses of endpoints, and the information on the best worker nodes that are forwarding nodes for the overloading situation of the local worker node. These variables can be used by the load-balancing function in Lines 26–37. This function aims to handle the requests locally as much as possible and forward overload requests to the best worker nodes. Once an incoming request arrives, the load-balancing function determines whether the local worker node has the available resource through a condition in Line 31. Note that the thresholds of the CPU and RAM can be set separately according to the processing time of the application, the system configuration, and user requirements (we set both thresholds to 90%, as will be discussed in Section 5 in detail). Then, if the local node still has sufficient resources, the request is forwarded to the backend pod at the local node. Otherwise, the request is forwarded to the backend pod in the best node selected before. In conclusion, *RAP* preferentially performs request-handling locally based on a list of local endpoints and monitors the application resources to make optimal load-balancing decisions.

We analyzed the time complexity of the RAP algorithm and obtained O(n) for n endpoints. Thus, we can state that the RAP algorithm is efficient as it does not degrade the load-balancing performance of the system. In detail, the endpoint collection and categorization in Lines 2–9 require n iterations to get EndpointMap in Line 6 because GetAppInfo(app) in Line 5 returns the endpoint and node information for each endpoint. The scoring process and best remote node selection process in Lines 10–24 also require n iterations because they need to calculate the resource score of the worker node including each endpoint. It is possible to repeat the scoring process for the same worker node more than once because one worker node may have one or more endpoints. However, considering the finite number of endpoints, this may not incur a significant computational overhead. Finally, the load balancer function in Lines 27–38 only requires O(1) to find the NextEndpoint. Thus, we can conclude that Algorithm 1 has a time complexity of O(n) for n endpoints.

## 5. Performance Evaluations

**Figure 4:**
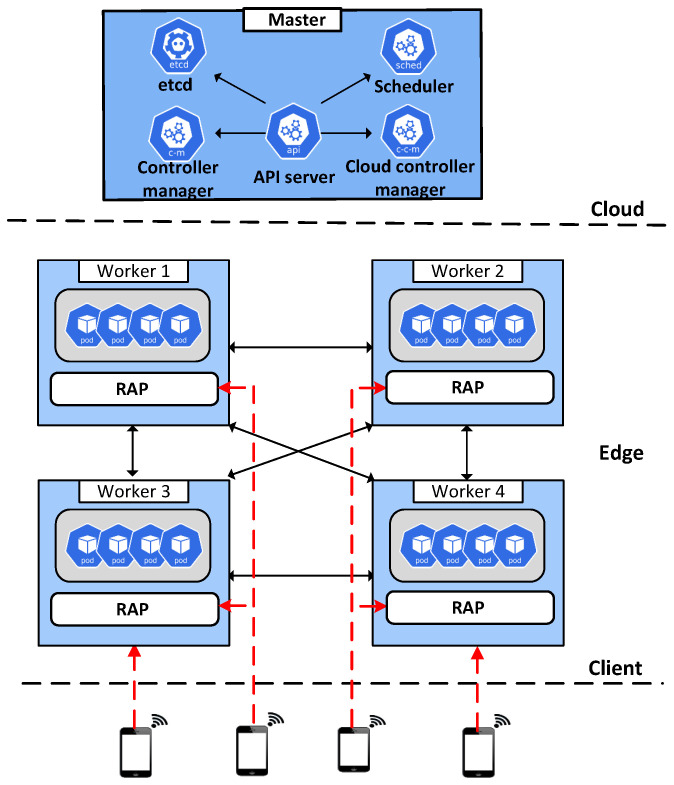
Architecture of *RAP* in an edge computing environment.

This section reports the evaluations conducted of the throughput and latency of application requests in a K8s-based edge computing environment, demonstrating the advantages of RAP. In the experimental edge computing environment, a cluster consisted of one master and four worker nodes with K8s version 19.03.8. The proposed *RAP* was implemented in each worker, as depicted in Figure 4. The master node was equipped with four CPU cores and 8 GB RAM, whereas each worker node had two CPU cores and 4 GB RAM. In addition, to create requests to send to the K8s cluster, we used the Apache HTTP server benchmarking (AB) tool [39] to create requests for worker nodes, as illustrated in Figure 4. AB also counts the number of completed requests per second and the time spent per request. We used the *NodePort Service* to receive incoming requests and set the overload threshold at 90%.

**Figure 5:**
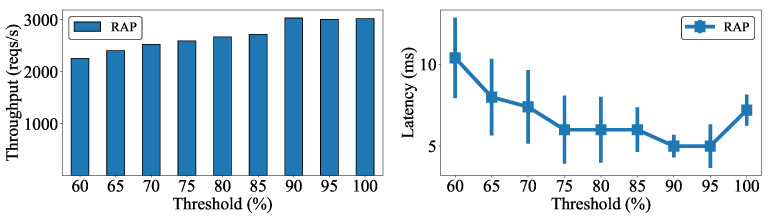
Effect of threshold on cluster performance.

To determine the appropriate threshold settings for the RAP algorithm, we evaluated the throughput and latency according to the threshold by sending requests to Worker 1 in the cluster. Figure 5 shows that both the throughput and latency improved as the threshold increased because the number of requests handled locally correspondingly increased. However, note that both metrics stop improving at a threshold of 90%, signifying that the bottleneck of resources of the local worker node has been reached. Thus, we concluded that 90% is the appropriate setting for the CPU and RAM thresholds, and we subsequently used this configuration for the diverse evaluations reported in this section.

We set up three experiments to evaluate the effect of a network delay on the application throughput, efficiency of the local processing, and *RAP* algorithm, with different request ratios. Note that *RAP* is implemented in *userspace*, so the achievable performance is fundamentally bounded by the operation of the *userspace* mode. However, we included the *iptables* mode, which works in kernel mode, in the comparison to demonstrate the efficiency of the proposed algorithm.

### 5.1. Effect of Delay between Worker Nodes on Cluster Performance

This subsection presents the analysis of the effect of delays between worker nodes on throughput and latency. We compared the performances of the *RAP*, *userspace*, and *iptables* proxy modes by varying the network delay between worker nodes from 3 to 18 ms. Furthermore, we set clients to send eight concurrent requests to each worker simultaneously.

**Figure 6:**
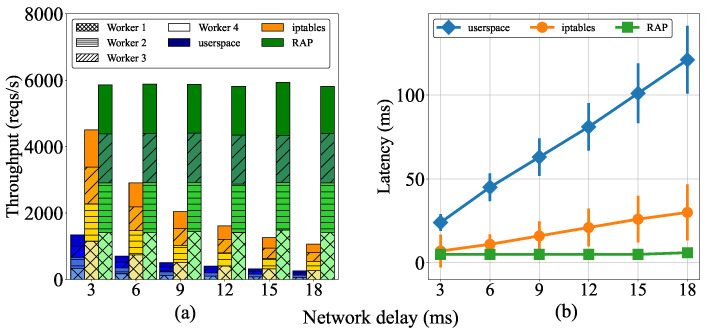
Cluster performance according to the network delay between workers: (**a**) throughput and (**b**) latency.

As shown in Figure 6a, the throughput of *iptables* was higher than that of *userspace* in all test cases. This is because the performance of *userspace* is limited by the bottleneck problem explained in Section 3.2. Moreover, we can observe that the throughputs of both *userspace* and *iptables* tended to decrease when the delays between the workers increased. For example, the throughputs of *iptables* and *userspace* were approximately 4500 and 1300 reps/s, respectively, when the network latency was set at 3 ms but both decreased to approximately 1000 and 220 reps/s, respectively, when the delay was increased to 18 ms. This is because both *userspace* and *iptables* evenly distribute the incoming requests to pods in the cluster, leading to the significant increase in the processing times of requests by the forwarding process. The throughput is calculated as the average number of client requests that can be processed within one second. Therefore, the throughput cannot increase if the processing times for requests are long. By contrast, the throughput in the *RAP* mode tends to be constant regardless of increasing network delay, and it remained stable at approximately 6000 reqs/s, as shown in Figure 6. This is because the requests are handled locally as much as possible instead of being forwarded to other worker nodes. Therefore, the effect of delay between worker nodes on the load-balancing operation was significantly reduced under *RAP*. In addition, if a local worker needs to offload requests, *RAP* forwards them to the best node to minimize the latency during load-balancing.

The latency of the three *kube-proxy* modes in Figure 6b was measured with the average latency based on forwarding and processing times. Notably, the latency of *RAP* was constant at approximately 5 ms. Meanwhile, the network latency of *userspace* increased from 25 to 120 ms as the delay increased, and *iptables* showed a moderate increase from 5 to >25 ms. Further, the latency-increase trend of *userspace* was higher than that of *iptables*. This is because the latency of the *iptables* mode originated solely from the forwarding process, whereas the latency of *userspace* was generated by both the forwarding process and transfers to user space by the operating system.

The results show that the load-balancing process is affected by environments where there are network delays between nodes. This generates latency for the services, which results in the degradation of the application QoS. Consequently, it is important to consider the delay between worker nodes, as it can significantly affect performance, especially in edge computing environments. Thereby, the *RAP* algorithm has demonstrated its advantages by ensuring stable performance and minimizing the request-handling latency.

### 5.2. Effect of Client Requests on Cluster Performance

In this section, we analyze the effect of client requests on the cluster performance in terms of throughput, latency, and application resource on workers. We evaluated the effect of diverse request scenarios based on two request patterns: concentrated and distributed. To create concentrated requests, we set a range of concurrent requests for one worker node of the cluster increasing from 1 to 32, as shown in test cases 1–6 of Table 2, while the other workers were ready to receive the forwarded requests. The distributed requests had different ratios for all workers, as shown in test cases 7–16 of Table 2, where the ratios represent the numbers of client requests accessing the service.

**Figure 7:**
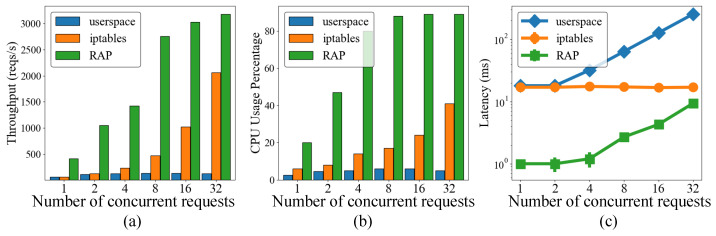
Effect of centralization requests on the cluster performance: (**a**) throughput, (**b**) latency, and (**c**) application resources.

Figure 7 illustrates the evaluation results of the concentrated requests in test cases 1–6, including the throughput, latency, and CPU usage percentage of Worker 1. As shown in Figure 7a, the throughput of *userspace* tended to increase to 130 reqs/s with four concurrent requests but it was bounded under more concurrent requests. Meanwhile, the throughput of *iptables* increased proportionally with the number of concurrent requests. It is clearly illustrated that when we increased the number of requests from 1 to 32, the throughput of Worker 1 considerably increased from approximately 120 reqs/s to >2000 reqs/s. Meanwhile, *RAP* showed a definite increasing trend of throughput from 500 reqs/s to 3100 reqs/s, which is higher than that under other modes. When the number of concurrent requests increased from one to four, all requests were handled locally and the throughput significantly increased from approximately 400 reqs/s to 1400 reqs/s. *RAP* forwarded the overloaded requests to the best worker when the number of requests was increased from 4 to 32; hence, the throughput increased from 1400 res/s to >3000 reqs/s. Therefore, this throughput is the sum of both local and remote worker processing. Note that the CPU usage in Figure 7b shows a similar trend to the throughput in Figure 7a.

We evaluated the QoS of the service of the application through the average latency in Figure 7c. In the figure, the latency of *userspace* tends to increase sharply during evaluation, starting at 17 ms and increasing to >150 ms. The latency of *iptables* remains stable at 17 ms. By contrast, *RAP* shows the lowest latency regardless of the number of concurrent requests. When the number of concurrent requests increased from one to four, the latency of *RAP* increased slightly from 1 ms to >2 ms because they were handled by Worker 1 locally. The average latency tended to increase when some of the requests were forwarded to another node when the number of requests increased from 4 to 32. However, the latency of *RAP* is lower than that of *userspace* and *iptables*. This is because the best remote worker to handle the overloaded requests is selected by the *RAP* algorithm. Moreover, the high latency of *userspace* comes from both delays in the network connection and the bottleneck limitation. *iptables* shows a steady trend because it works in kernel space, which is faster than userspace.

**Figure 8:**
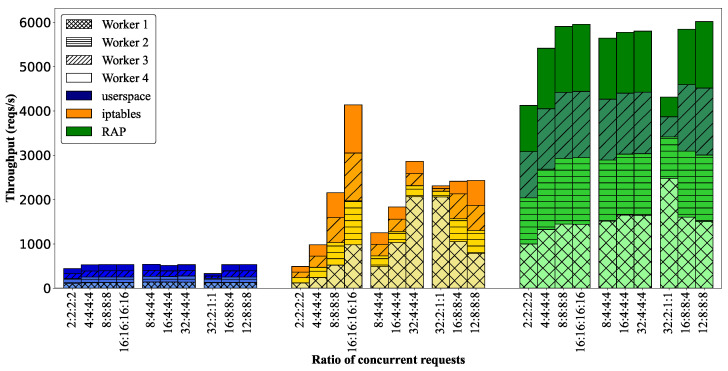
Total throughput of cluster in the distributed request experiment.

We also analyzed the effect of distributed requests on performance by evaluating the throughput of the various request distributions listed for test cases 7–16 in Table 2. As shown in Figure 8, *userspace* has bounded performance, although requests are distributed to all workers. The total throughput in the *iptables* mode changes based on the distribution of requests. This throughput trend is the same with concentrated request test cases. The figure shows that *RAP* can achieve a high throughput under most request ratios. In test cases 7–10, all workers receive an equal ratio, and the number of concurrent requests increases from 2 to 16. The total throughput of the cluster significantly increases from 4100 req/s to >6000 req/s, and we can see that the maximum capacity of the *RAP* algorithm is represented by an 8:8:8:8 ratio.

In test cases 11–13, we set an imbalance of requests to ensure that approximately 80% of the resources of remote workers were used by four concurrent requests, whereas the number of concurrent requests at a local worker increased from 8 to 32. Figure 8 illustrates a slightly increasing tendency of the total throughput from 5640 to 5800 reqs/s. This is because the remote workers do not have sufficient resources to handle the forwarded requests. Because *RAP* cannot identify any of the best nodes to which the overloaded requests can be offloaded in this situation, it continues to handle requests locally. We can conclude that *RAP* does not create stressful requests for other overloaded nodes in the cluster.

In test cases 14–16 in Table 2, we set the ratio of requests differently while keeping the total number of requests the same. The total throughputs for these test cases were different. For example, the cluster had a maximum capacity of 6000 reqs/s with a 12:8:8:8 ratio, but it only achieved approximately 4200 reqs/s with a 32:2:1:1 ratio. We can observe that *RAP* has the highest performance if it can handle as many requests locally at each worker as possible. For example, 32 concurrent requests in the 32:2:1:1 case are too much to handle for Worker 1, so a portion of the requests must be forwarded to other nodes. By contrast, the 16:8:8:4 and 12:8:8:8 cases show an increase in throughput of approximately 25% because the number of concurrent requests handled locally for each worker increased. Therefore, we can conclude that *RAP* can work efficiently with both concentrated and distributed requests, increasing the throughput and reducing latency based on local handling and optimal forwarding of requests.

## 6. Conclusions

In this paper, we proposed an improved proxy called *RAP* for K8s-based edge computing environments. *RAP* preferentially performs request-handling locally instead of evenly distributing them. In addition, *RAP* provides the ability to monitor the resource status of workers during the load-balancing operation. The best node is selected through the monitoring process to offload requests for a local worker when it becomes overloaded. Our performance evaluation results demonstrated that *RAP* significantly improved both the throughput and latency regardless of the number of concentrated and distributed requests and that *RAP* was efficient in high-delay environments. In future work, we will expand the *RAP* algorithm to monitor other cluster resources such as graphics processing units and storage. Moreover, we will investigate the application of *RAP* in other containerized orchestrations.

## Figures and Tables

**Table 1:** Key notation.

Notation	Description
α	Application deployed in edge computing
*r*	Resource parameter including CPU, RAM, and latency
*N*	Set of remote workers
*i*	Individual remote worker, where iϵN
$R_{r}^{i}$	Resource *r* of worker *i*
$\beta_{r}^{i}$	Individual denominator of resource *r* for worker *i*
$W_{r}$	Weight factor of resource *r*
$\tau_{i}$	Score of each worker node
$\tau_{BestNode}$	Score of the best worker node

**Table 2:** Ratio type.

Test Cases	Requests	Note	Ratio Worker 1:Worker 2:Worker 3:Worker 4
1–6	Concentrated requests		[1, 2, 4, 8, 16, 32]:0:0:0
7	Distributed requests	Balance requests	2:2:2:2
8			4:4:4:4
9			8:8:8:8
10			16:16:16:16
11		Imbalance requests	8:4:4:4
12			16:4:4:4
13			32:4:4:4
14		Equal total throughput	32:2:1:1
15			16:8:8:4
16			12:8:8:8
